# Synthesis, crystal structure and Hirshfeld surface analysis of a cadmium complex of naphthalene-1,5-di­sulfonate and *o*-phenyl­enedi­amine

**DOI:** 10.1107/S2056989023010125

**Published:** 2023-11-30

**Authors:** Jabbor Suyunov, Batirbay Torambetov, Khayit Turaev, Shakhnoza Kadirova, Bekmurod Alimnazarov, Jamshid Ashurov

**Affiliations:** a Termez State University, Barkamol Avlod St. 43, Termez, 190111, Uzbekistan; b National University of Uzbekistan named after Mirzo Ulugbek, 4 University St, Tashkent, 100174, Uzbekistan; c Institute of Bioorganic Chemistry, Academy of Sciences of Uzbekistan, M. Ulugbek St. 83, Tashkent, 100125, Uzbekistan; Universidad de Los Andes Mérida, Venezuela

**Keywords:** crystal structure, cadmium complex, *o*-phenyl­enedi­amine, naphthalene-1,5-di­sulfonate, Hirshfeld surface analysis

## Abstract

A novel *o*-phenyl­enedi­amine (opda)-based cadmium complex, bis­(benzene-1,2-di­amine-κ^2^ *N*,*N*′)bis­(benzene-1,2-di­amine-κ*N*)cadmium(II) naphthalene-1,5-di­sulfonate, [Cd(C_6_H_8_N_2_)_4_](C_10_H_6_O_6_S_2_), was synthesized. The complex salt crystallizes in the monoclinic space group *C*2/*c*. The Cd atom occupies a special position and coordinates six nitro­gen atoms from four *o*-phenyl­enedi­amine mol­ecules, two as chelating ligands and two as monodentate ligands. The amino H atoms of opda inter­act with two O atoms of the naphthalene-1,5-di­sulfonate anions. The anions act as bridges between [Cd(opda)_4_]^2+^ cations, forming a two-dimensional network in the [010] and [001] directions. The Hirshfeld surface analysis shows that the primary factors contributing to the supramolecular inter­actions are short contacts, particularly van der Waals forces of the type H⋯H, O⋯H and C⋯H.

## Abstract 2

The mol­ecular and crystal structure of a cadmium complex of naphthalene-1,5-di­sulfonate and *o*-phenyl­enedi­amine was studied and Hirshfeld surfaces and fingerprint plots were generated to investigate the various inter­molecular inter­actions.

## 1. Chemical context

Cadmium is widely used in the fabrication of rechargeable batteries, in alloys, coatings (electroplating), solar cells, plastic stabilizers, phosphate fertilizers, and pigments (Omar *et al.*, 2014 ▸; Morrow, 2010 ▸; Indumathi *et al.*, 2011 ▸; Kapadnis *et al.*, 2020 ▸; Wakkaf *et al.*, 2020 ▸; Roberts, 2014 ▸; Cesaratto *et al.*, 2014 ▸). Given its common use, cadmium is now spreading widely in the environment (Kumar *et al.*, 2019 ▸; Wang *et al.*, 2023 ▸) and, due to its toxicity, it is necessary to prevent the technogenic spread of cadmium and its harmful consequences.

When it comes to complex formation, organic ligands with multiple donor centers that form chelates play a crucial role. Stable complexes are obtained by the formation of a ring consisting of five or six members, including a metal atom in the ring. Additionally, when the bidentate ligand is involved in coordination with the central atom by forming a five-membered ring, it further increases the stability of the complex (Lawrance, 2010 ▸). The conformational change of five- and six-membered di­amine chelate rings in metal complexes has been thoroughly documented (Corey *et al.*, 1959 ▸; Gollogly *et al.*, 1967 ▸; Ma *et al.*, 2005 ▸, 2012 ▸). In this regard, the *o*-phenyl­enedi­amine (opda) ligand has been extensively studied as a linking agent that effectively forms a chelating ring with a variety of metal cations. Developing metal ion sorbents utilizing these organic ligands is both economically and practically efficient. We present a report on the crystal structure and Hirshfeld surface analysis of a newly synthesized Cd complex salt of naphthalene-1,5-di­sulfonate with *o*-phenyl­enedi­amine (opda) as its base. 
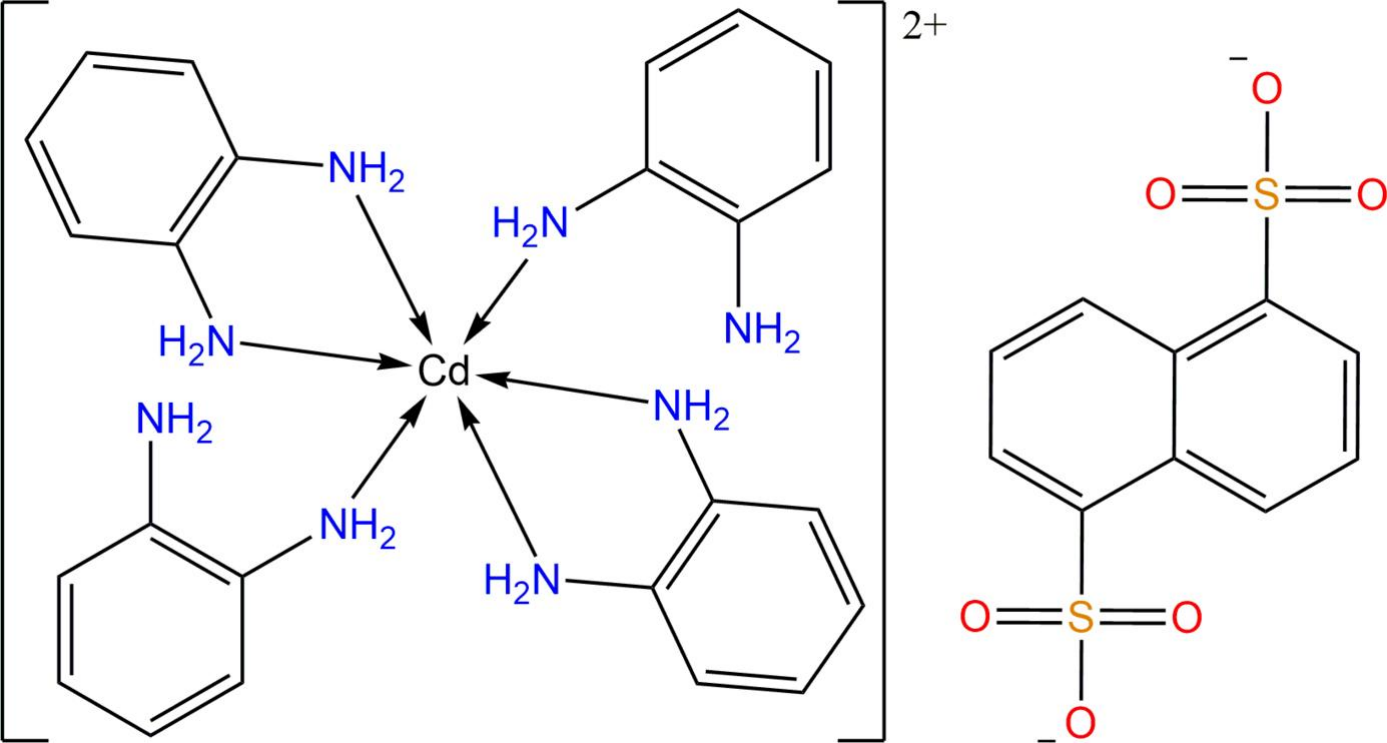


## 2. Structural commentary

The complex salt [Cd(opda)_4_](C_10_H_6_O_6_S_2_) crystallizes in the monoclinic system, space group *C*2/*c*. The Cd atom occupies a special position with twofold symmetry (Wyckoff position 4*e*). The midpoint of the naphthalene-1,5-di­sulfonate anion lies on a center of inversion (Wyckoff position 4*b*). Therefore, the asymmetric unit consists of one half of the complex cation and anion.

**Figure 1:**
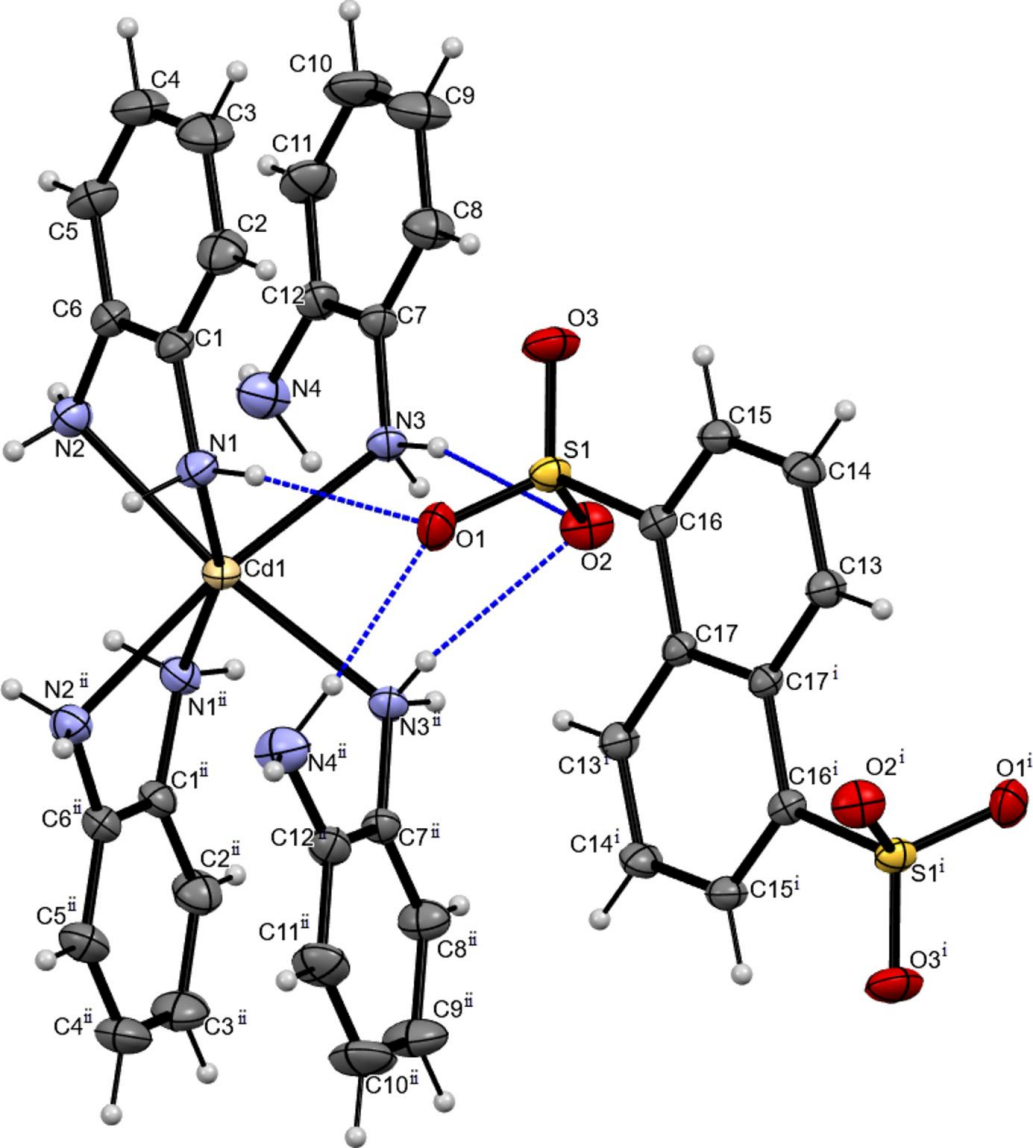
Mol­ecular structure of the title compound. The hydrogen bond is indicated by a dashed line. Displacement ellipsoids are plotted at the 30% probability level. [Symmetry codes: (i) 1 − *x*, 2 − *y*, 1 − *z*; (ii) 1 − *x*, *y*, 3/2 - *z.*]

Fig. 1 ▸ shows the coordination environment of the Cd atom and the hydrogen bonds between the amine hydrogens and the oxygen atoms of the anion. The Cd atom coordinates six nitro­gen atoms which come from two *o*-phenyl­enedi­amine mol­ecules and their two symmetry-related counterparts [symmetry operation: (i) 1 − *x*, *y*, 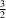 − *z*]. The naphthalene-1,5-di­sulfonate anion is completed by atoms related by 1 − *x*, 2 − *y*, 1 − *z* [symmetry operation: (ii)]. Two of the *o*-phenyl­enedi­amine ligands are coordinated in a chelating fashion while the other two form monodentate bonds. The chelating and monodentate ligands are located in *cis* positions. The complex exhibits a distorted octa­hedral coordination sphere for the metal atom due to the reduction of the N1—Cd1—N2 angle [70.41 (6)°]. This value is similar to those found in other cadmium complexes reported by several authors (Gonzalez Guillen *et al.*, 2018 ▸; Malinina *et al.*, 2007 ▸; Rahman *et al.*, 2017 ▸; Supriya, 2009 ▸) where a chelate ring is observed. The largest bond angle between atoms in the basal plane in this polyhedron is 101.57 (6)° for N1^i^—Cd1—N3. Distortions are also observed in the angles between opposite vertex atoms. A value of 162.11 (10)° is observed for N1^i^—Cd1—N1 and 170.30 (6)° for N2—Cd1—N3^i^ and N2^i^—Cd1—N3. All Cd1—N bonds have very close values with the maximum difference of only 0.0842 Å. The chelating coordination mode slightly affects the positions of the N and C atoms in the opda ligands. The opda ligands are approximately planar, with a maximum deviation from the least-squares plane of 0.003 Å for atom C12 in the monodentate one (r.m.s. deviation 0.002 Å) and 0.005 Å for atom C1 in the bidentate one (0.002 Å r.m.s. deviation). The dihedral angle between the main planes of the phenyl ring (C1–C6 or C7–C12) and the N—C—C—N fragment is 4.16 (12)° in the bidentate ligand and 1.73 (13)° in the monodentate ligand.

## 3. Supra­molecular features

**Figure 2:**
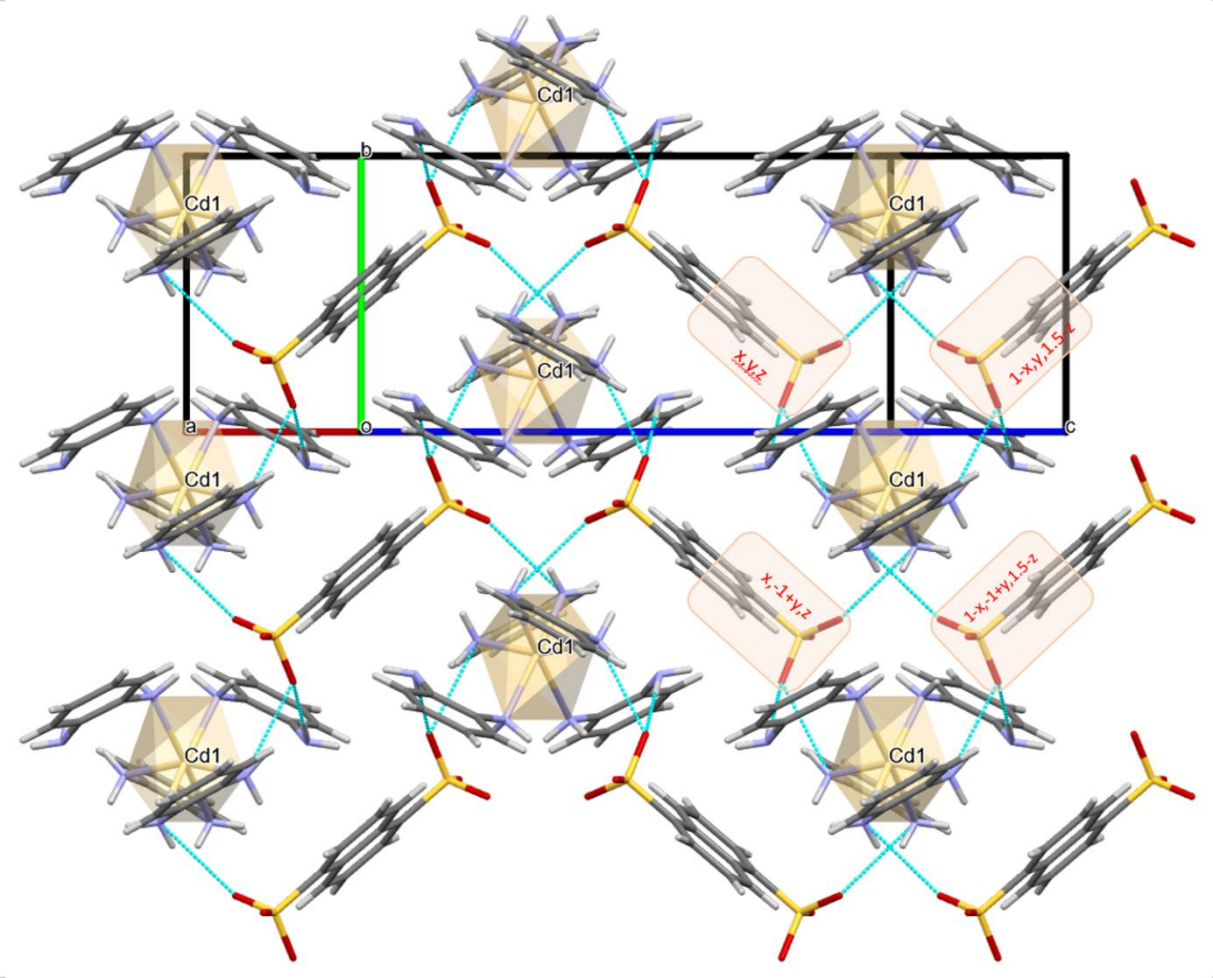
Crystal packing of the corrugated layers parallel to the (200) plane showing hydrogen bonds in cyan lines. Projection along the perpendicular to the (200) plane.

In the crystal, the [Cd(opda)_4_]^2+^ cation and the naphthalene-1,5-di­sulfonate dianion inter­act *via* N1—H1*B*⋯O1, N3—H3*A*⋯O2, N3—H3*B*⋯O2^ii^, N4—H4*A*⋯O1^ii^ and N2—H2*A*⋯O2^i^ hydrogen bonds (Fig. 1 ▸, Table 1 ▸). Here the O1 atom participates in a bifurcated hydrogen bond with N1 and N4^ii^ and the O2 atom does the same with atoms N3 and N3^ii^. These hydrogen bonds form infinite two-dimensional networks along the [010] and [001] directions in which the naphthalene-1,5-di­sulfonate dianions serve as bridges between [Cd(opda)_4_]^2+^ cations as hydrogen bond acceptors in both directions (Fig. 2 ▸).

Each [Cd(opda)_4_]^2+^ cation is surrounded by naphthalene-1,5-di­sulfonate^2−^ anions from four positions, and their oxygen atoms (symmetry codes *x, y, z*; 1 - *x, y*, 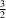 − *z*; 1 − *x*, −1 + *y*, 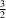 − *z*; *x*, −1 + *y*, *z*) are hydrogen-bonded to the NH groups of the cation. These hydrogen bonds serve to grow the network along the [010] direction (Fig. 2 ▸). At the same time, the naphthalene-1,5-di­sulfonate anions are attached to neighboring [Cd(opda)_4_]^2+^ cations, through hydrogen bonds that ensure the growth of the crystal network in the [001] direction (Figs. 2 ▸ and 3 ▸).

## 4. Hirshfeld surface analysis

**Figure 4:**
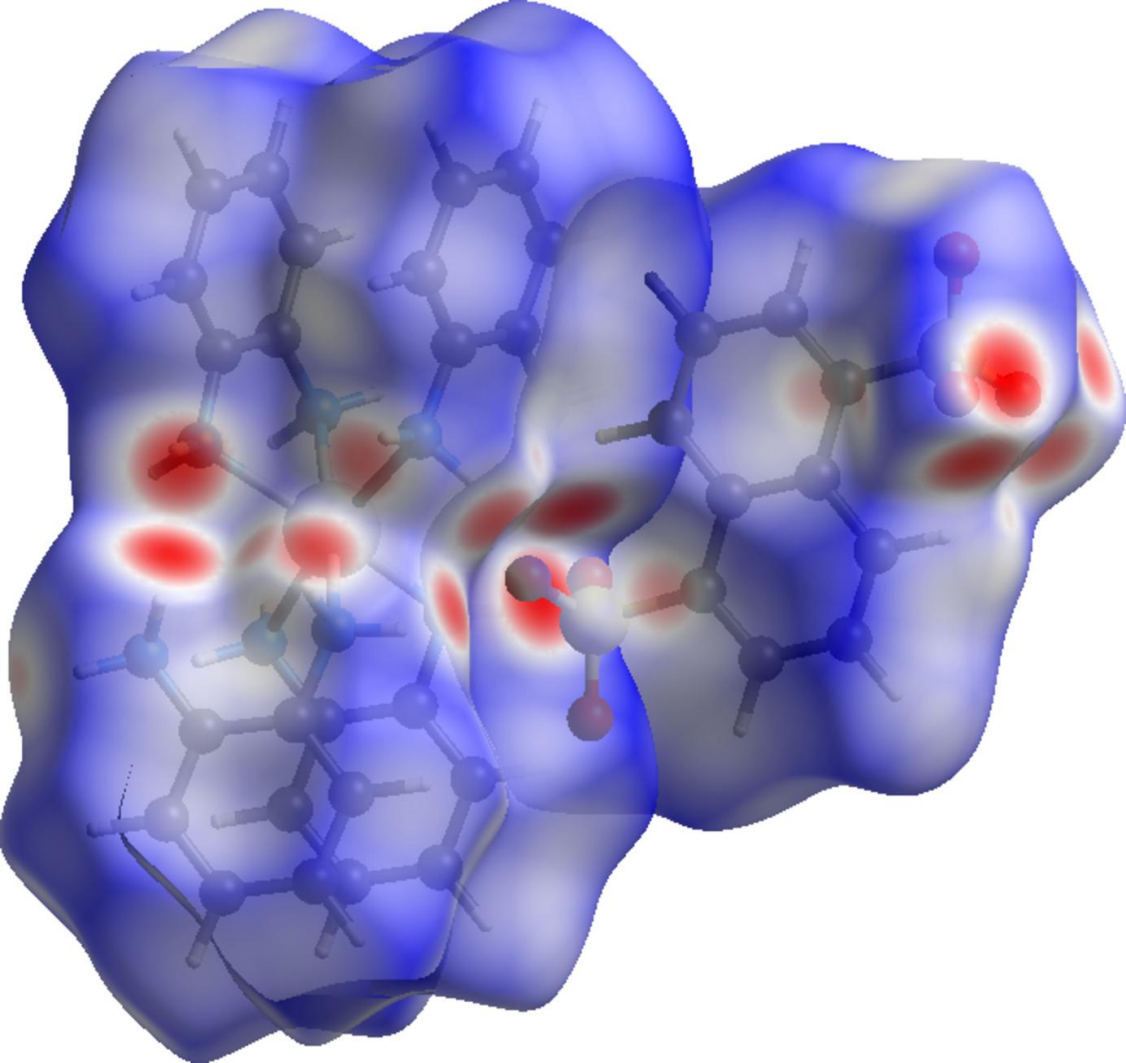
Hirshfeld surfaces of the [Cd(opda)_4_]^2+^ cation and the [naphthalene-1,5-di­sulfonate]^2−^ anion mapped with *d*_norm_.

To further investigate the inter­molecular inter­actions present in the title compound, a Hirshfeld surface analysis (Spackman & Byrom, 1997 ▸) was performed and the two-dimensional fingerprint plots were generated with *CrystalExplorer17* (Spackman *et al.*, 2021 ▸). The Hirshfeld surfaces mapped over *d*_norm_ for both moieties (representing various inter­actions with the default colors) are shown in Fig. 4 ▸. The default scaling was used {−0.4573, 1.2430 Å for the [Cd(opda)_4_] cation and −0.4579, 1.0829 Å for the naphthalene-1,5-di­sulfone anion}.

**Figure 5:**
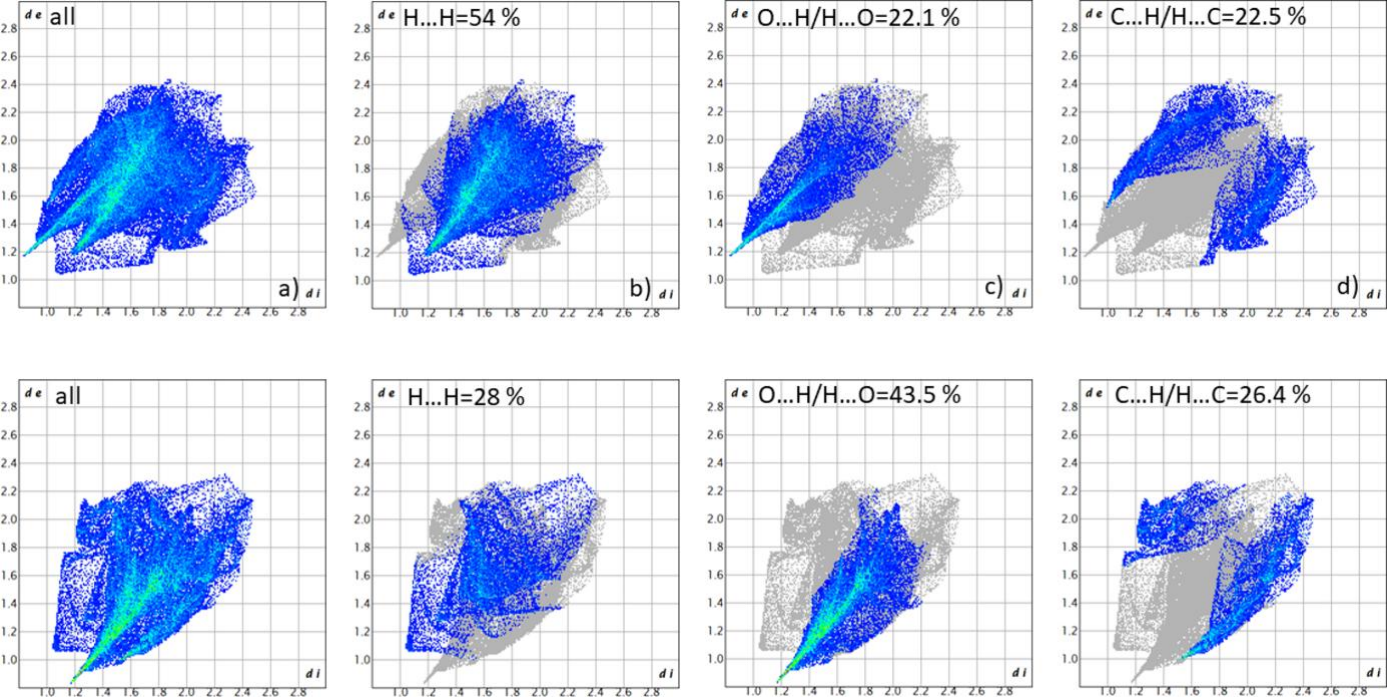
Contributions of the various contacts to the two-dimensional fingerprint plots built using the Hirshfeld surfaces of the [Cd(opda)_4_]^2+^ cation (at the top) and the [naphthalene-1,5-di­sulfonate]^2−^ anion (at the bottom).

The two-dimensional (2D) fingerprint plots (McKinnon *et al.*, 2007 ▸) are shown in Fig. 5 ▸. The most significant inter­actions, whose contribution to the Hirshfeld surface area exceed 20.0% at least for one of the ions in the structure, are H⋯H {54% and 28% for the [Cd(opda)_4_] cation and naphthalene-1,5-di­sulfone anion moieties, respectively}, H⋯O/O⋯H (22.1% and 43.5%) and C⋯H/H⋯C (22.5% and 26.4%). These inter­actions play a crucial role in the overall consolidation of the crystal structure.

## 5. Database survey

A survey of the Cambridge Structural Database (CSD, version 5.43, update of November 2021; Groom *et al.*, 2016) revealed that 74 crystal structures have been reported for chelate complexes of *o*-phenyl­enedi­amine with several metal atoms. The CSD includes structures of complexes of Ni^II^ and Cr^II^ based on *o*-phenyl­enedi­amine with ratios of 1:4 and six-coordination numbers (OPDANI, Elder *et al.*, 1974 ▸; FENVOK, Ariyananda *et al.*, 2005 ▸; SOFXIU, Jubb *et al.*, 1991 ▸). However, no co-crystal complexes and metal complexes containing *o*-phenyl­enedi­amine and the naphthalene-1,5-di­sulfonate anion together in the crystal have been reported.

## 6. Synthesis and crystallization

Ethanol/water 1:1 (10 mL) solutions of Cd(CH_3_COO)_2_·2H_2_O (0.266 g, 0.001 mol) and sodium naphthalene-1,5-di­sulfonate (0.332 g, 0.001 mol) were combined. To the obtained solution, a 10 ml ethanol solution of *o*-phenyl­enedi­amine (opda) (0.432 g, 0.004 mol) was added dropwise and then stirred at 323 K for 30 minutes. The final solution was left to crystallize and X-ray quality single crystals were produced after 15 days by slow evaporation of the solvent.

## 7. Refinement

Crystal data, data collection and structure refinement details are summarized in Table 2 ▸. All the hydrogen atoms were located in difference-Fourier maps and refined isotropically.

## Supplementary Material

Crystal structure: contains datablock(s) I. DOI: 10.1107/S2056989023010125/dj2067sup1.cif

Structure factors: contains datablock(s) I. DOI: 10.1107/S2056989023010125/dj2067Isup2.hkl

CCDC reference: 2257108

Additional supporting information:  crystallographic information; 3D view; checkCIF report

## Figures and Tables

**Figure 3:**
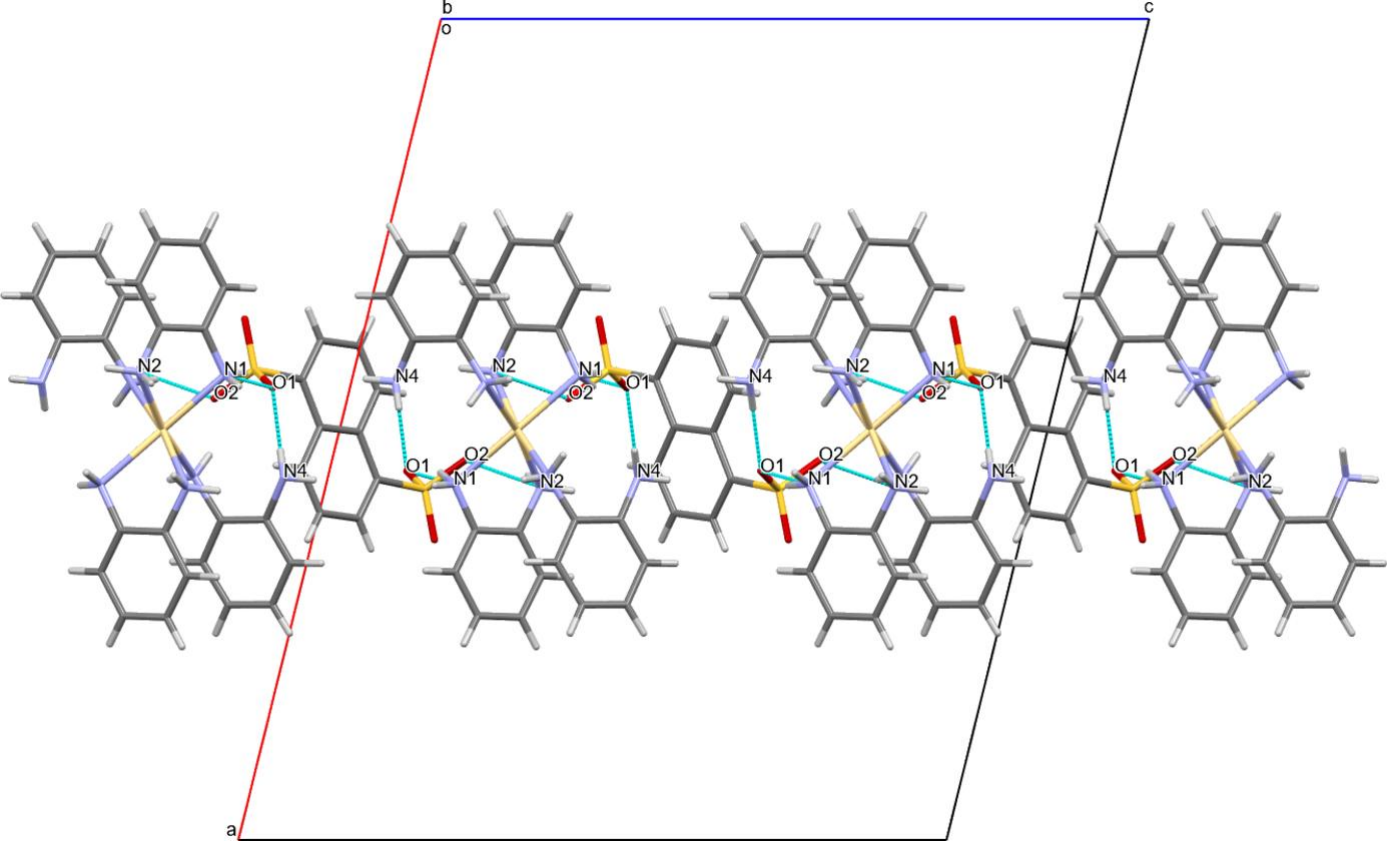
View of the mol­ecular packing showing the hydrogen-bonding inter­actions that extend along the *c-*axis direction.

**Table 1:** Hydrogen-bond geometry (Å, °)

*D*—H⋯*A*	*D*—H	H⋯*A*	*D*⋯*A*	*D*—H⋯*A*
N3—H3*A*⋯O2	0.86 (3)	2.36 (3)	3.185 (2)	160 (2)
N2—H2*A*⋯O2^i^	0.81 (3)	2.30 (3)	3.046 (3)	154 (3)
N1—H1*B*⋯O1	0.83 (3)	2.18 (3)	2.955 (2)	156 (3)
N3—H3*B*⋯O2^ii^	0.81 (3)	2.37 (3)	3.097 (2)	150 (2)
N4—H4*A*⋯O1^ii^	0.91 (3)	2.11 (3)	3.021 (3)	176 (3)

Symmetry codes: (i) 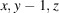 ; (ii) 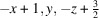 .

**Table 2:** Experimental details

Crystal data	
Chemical formula	[Cd(C_6_H_8_N_2_)_4_](C_10_H_6_O_6_S_2_)
*M*_r_	831.24
Crystal system, space group	Monoclinic, *C*2/*c*
Temperature (K)	293
*a*, *b*, *c* (Å)	23.5743 (2), 7.7286 (1), 19.7260 (2)
β (°)	103.858 (1)
*V* (Å^3^)	3489.39 (7)
*Z*	4
Radiation type	Cu *K*α
μ (mm^−1^)	6.62
Crystal size (mm)	0.3 × 0.26 × 0.2

Data collection	
Diffractometer	XtaLAB Synergy, Single source at home/near, HyPix3000
Absorption correction	Multi-scan (*CrysAlis PRO*; Rigaku OD, 2023)
*T*_min_, *T*_max_	0.698, 1.000
No. of measured, independent and observed [*I* > 2σ(*I*)] reflections	16542, 3390, 3298
*R*_int_	0.030
(sin θ/λ)_max_ (Å^−1^)	0.615

Refinement	
*R*[*F* ^2^ > 2σ(*F* ^2^)], *wR*(*F* ^2^), *S*	0.023, 0.061, 1.04
No. of reflections	3390
No. of parameters	264
H-atom treatment	H atoms treated by a mixture of independent and constrained refinement
Δρ_max_, Δρ_min_ (e Å^−3^)	0.29, −0.51

Computer programs: *CrysAlis PRO* (Rigaku OD, 2022 ▸), *SHELXT2018/2* (Sheldrick, 2015 ▸a), *SHELXL2018/3* (Sheldrick, 2015 ▸b) and *OLEX2* (Dolomanov *et al.*, 2009 ▸).

## supplementary crystallographic information

### Crystal data

**Table:**

[Cd(C_6_H_8_N_2_)_4_](C_10_H_6_O_6_S_2_)	*F*(000) = 1704
*M**_r_* = 831.24	*D*_x_ = 1.582 Mg m^−^^3^
Monoclinic, *C*2/*c*	Cu *K*α radiation, λ = 1.54184 Å
*a* = 23.5743 (2) Å	Cell parameters from 13387 reflections
*b* = 7.7286 (1) Å	θ = 3.9–71.3°
*c* = 19.7260 (2) Å	µ = 6.62 mm^−^^1^
β = 103.858 (1)°	*T* = 293 K
*V* = 3489.39 (7) Å^3^	Block, colourless
*Z* = 4	0.3 × 0.26 × 0.2 mm

### Data collection

**Table:**

XtaLAB Synergy, Single source at home/near, HyPix3000 diffractometer	3390 independent reflections
Radiation source: micro-focus sealed X-ray tube, PhotonJet (Cu) X-ray Source	3298 reflections with *I* > 2σ(*I*)
Mirror monochromator	*R*_int_ = 0.030
Detector resolution: 10.0000 pixels mm^-1^	θ_max_ = 71.4°, θ_min_ = 3.9°
ω scans	*h* = −23→28
Absorption correction: multi-scan (CrysAlisPro; Rigaku OD, 2023)	*k* = −9→9
*T*_min_ = 0.698, *T*_max_ = 1.000	*l* = −24→24
16542 measured reflections	

### Refinement

**Table:**

Refinement on *F*^2^	Hydrogen site location: mixed
Least-squares matrix: full	H atoms treated by a mixture of independent and constrained refinement
*R*[*F*^2^ > 2σ(*F*^2^)] = 0.023	*w* = 1/[σ^2^(*F*_o_^2^) + (0.0374*P*)^2^ + 1.6331*P*] where *P* = (*F*_o_^2^ + 2*F*_c_^2^)/3
*wR*(*F*^2^) = 0.061	(Δ/σ)_max_ = 0.001
*S* = 1.04	Δρ_max_ = 0.29 e Å^−^^3^
3390 reflections	Δρ_min_ = −0.51 e Å^−^^3^
264 parameters	Extinction correction: *SHELXL2018/3* (Sheldrick, 2015b), Fc^*^=kFc[1+0.001xFc^2^λ^3^/sin(2θ)]^-1/4^
0 restraints	Extinction coefficient: 0.00060 (3)
Primary atom site location: dual	

### Special details

**Table:**

Geometry. All esds (except the esd in the dihedral angle between two l.s. planes) are estimated using the full covariance matrix. The cell esds are taken into account individually in the estimation of esds in distances, angles and torsion angles; correlations between esds in cell parameters are only used when they are defined by crystal symmetry. An approximate (isotropic) treatment of cell esds is used for estimating esds involving l.s. planes.

### Fractional atomic coordinates and isotropic or equivalent isotropic displacement parameters (Å^2^)

**Table:**

	*x*	*y*	*z*	*U*_iso_*/*U*_eq_	
Cd1	0.500000	0.30368 (2)	0.750000	0.03390 (8)	
S1	0.57283 (2)	0.75940 (6)	0.64093 (2)	0.03278 (11)	
O3	0.63434 (6)	0.7592 (2)	0.67223 (8)	0.0538 (4)	
O2	0.53752 (7)	0.82032 (19)	0.68767 (7)	0.0461 (4)	
O1	0.55070 (7)	0.59515 (18)	0.60921 (8)	0.0471 (3)	
N3	0.55688 (7)	0.5428 (2)	0.81048 (8)	0.0324 (3)	
N1	0.56647 (7)	0.2566 (2)	0.68088 (9)	0.0359 (3)	
N2	0.57034 (7)	0.0874 (2)	0.80405 (10)	0.0412 (4)	
C17	0.50295 (7)	0.9405 (2)	0.52814 (8)	0.0264 (3)	
C7	0.61049 (7)	0.5314 (2)	0.86432 (8)	0.0320 (4)	
N4	0.55812 (9)	0.3778 (3)	0.93793 (11)	0.0553 (5)	
C15	0.60837 (7)	0.9911 (2)	0.55757 (9)	0.0334 (4)	
H15	0.645358	0.968869	0.585633	0.040*	
C16	0.56084 (7)	0.9092 (2)	0.57062 (8)	0.0277 (3)	
C13	0.54760 (8)	1.1410 (3)	0.46009 (9)	0.0341 (4)	
H13	0.543889	1.218105	0.423086	0.041*	
C14	0.60174 (8)	1.1089 (3)	0.50191 (10)	0.0382 (4)	
H14	0.634285	1.165022	0.493536	0.046*	
C12	0.60984 (8)	0.4504 (3)	0.92721 (9)	0.0391 (4)	
C6	0.62457 (8)	0.1375 (2)	0.78897 (10)	0.0361 (4)	
C1	0.62273 (8)	0.2241 (2)	0.72671 (10)	0.0348 (4)	
C2	0.67355 (10)	0.2866 (3)	0.71257 (13)	0.0493 (5)	
H2	0.672048	0.345609	0.671084	0.059*	
C8	0.66153 (9)	0.5986 (3)	0.85278 (11)	0.0468 (5)	
H8	0.661275	0.653233	0.810709	0.056*	
C5	0.67805 (9)	0.1121 (3)	0.83592 (12)	0.0506 (5)	
H5	0.679763	0.052972	0.877417	0.061*	
C11	0.66247 (10)	0.4376 (3)	0.97790 (12)	0.0572 (6)	
H11	0.663070	0.383961	1.020270	0.069*	
C3	0.72678 (10)	0.2621 (4)	0.75990 (16)	0.0609 (6)	
H3	0.760948	0.304468	0.750326	0.073*	
C4	0.72872 (10)	0.1742 (3)	0.82129 (15)	0.0619 (7)	
H4	0.764382	0.156738	0.853024	0.074*	
C9	0.71343 (10)	0.5848 (4)	0.90398 (14)	0.0677 (7)	
H9	0.747926	0.630189	0.896406	0.081*	
C10	0.71319 (11)	0.5032 (4)	0.96591 (14)	0.0718 (8)	
H10	0.747871	0.492453	1.000044	0.086*	
H3A	0.5610 (10)	0.613 (4)	0.7782 (13)	0.049 (7)*	
H2A	0.5562 (12)	0.003 (4)	0.7824 (14)	0.063 (8)*	
H1A	0.5530 (11)	0.162 (4)	0.6578 (13)	0.051 (7)*	
H1B	0.5681 (12)	0.337 (4)	0.6538 (15)	0.064 (9)*	
H3B	0.5317 (11)	0.593 (3)	0.8244 (13)	0.054 (7)*	
H4A	0.5264 (13)	0.448 (4)	0.9240 (14)	0.064 (8)*	
H2B	0.5746 (12)	0.072 (4)	0.8486 (16)	0.071 (9)*	
H4B	0.5614 (16)	0.361 (5)	0.980 (2)	0.102 (13)*	

### Atomic displacement parameters (Å^2^)

**Table:**

	*U*^11^	*U*^22^	*U*^33^	*U*^12^	*U*^13^	*U*^23^
Cd1	0.02348 (11)	0.03120 (12)	0.04631 (12)	0.000	0.00697 (7)	0.000
S1	0.0289 (2)	0.0389 (2)	0.0286 (2)	0.00213 (17)	0.00313 (16)	0.00811 (16)
O3	0.0305 (7)	0.0741 (10)	0.0503 (9)	0.0027 (7)	−0.0035 (6)	0.0233 (8)
O2	0.0478 (8)	0.0612 (10)	0.0323 (7)	0.0017 (6)	0.0153 (6)	0.0050 (6)
O1	0.0549 (9)	0.0323 (7)	0.0501 (8)	0.0017 (6)	0.0044 (6)	0.0089 (6)
N3	0.0312 (8)	0.0332 (8)	0.0283 (7)	0.0007 (6)	−0.0019 (6)	0.0012 (6)
N1	0.0329 (8)	0.0379 (9)	0.0361 (8)	0.0067 (7)	0.0064 (6)	−0.0009 (7)
N2	0.0349 (8)	0.0400 (10)	0.0458 (10)	0.0000 (7)	0.0041 (7)	0.0097 (8)
C17	0.0245 (8)	0.0272 (8)	0.0277 (7)	0.0021 (6)	0.0070 (6)	0.0004 (6)
C7	0.0308 (9)	0.0319 (9)	0.0299 (8)	0.0015 (7)	0.0003 (6)	−0.0013 (7)
N4	0.0487 (11)	0.0661 (13)	0.0476 (11)	−0.0084 (10)	0.0048 (8)	0.0217 (10)
C15	0.0237 (8)	0.0391 (10)	0.0359 (9)	0.0002 (7)	0.0039 (6)	0.0026 (7)
C16	0.0265 (8)	0.0303 (8)	0.0259 (7)	0.0031 (6)	0.0059 (6)	0.0006 (6)
C13	0.0298 (8)	0.0371 (9)	0.0359 (9)	−0.0002 (7)	0.0091 (7)	0.0108 (7)
C14	0.0251 (8)	0.0428 (11)	0.0475 (10)	−0.0044 (7)	0.0103 (7)	0.0090 (8)
C12	0.0384 (10)	0.0393 (10)	0.0351 (9)	0.0002 (8)	0.0001 (7)	0.0038 (8)
C6	0.0292 (9)	0.0315 (9)	0.0449 (10)	0.0043 (7)	0.0035 (7)	0.0007 (8)
C1	0.0274 (9)	0.0344 (9)	0.0421 (10)	0.0066 (7)	0.0072 (7)	−0.0038 (7)
C2	0.0404 (11)	0.0536 (13)	0.0580 (13)	0.0055 (9)	0.0196 (10)	0.0023 (10)
C8	0.0370 (10)	0.0579 (13)	0.0454 (11)	−0.0042 (9)	0.0096 (8)	0.0041 (9)
C5	0.0380 (11)	0.0556 (14)	0.0517 (12)	0.0084 (9)	−0.0022 (9)	0.0069 (10)
C11	0.0511 (13)	0.0697 (15)	0.0404 (11)	0.0033 (11)	−0.0091 (9)	0.0125 (10)
C3	0.0315 (11)	0.0721 (16)	0.0802 (18)	−0.0003 (11)	0.0158 (11)	−0.0033 (14)
C4	0.0299 (11)	0.0720 (17)	0.0753 (17)	0.0078 (10)	−0.0039 (10)	−0.0017 (13)
C9	0.0311 (11)	0.093 (2)	0.0735 (16)	−0.0103 (12)	0.0026 (10)	0.0021 (15)
C10	0.0386 (12)	0.098 (2)	0.0636 (16)	0.0009 (13)	−0.0183 (11)	0.0066 (14)

### Geometric parameters (Å, º)

**Table:**

Cd1—N3^i^	2.4230 (15)	N4—H4B	0.82 (4)
Cd1—N3	2.4230 (15)	C15—H15	0.9300
Cd1—N1^i^	2.3388 (16)	C15—C16	1.364 (2)
Cd1—N1	2.3388 (16)	C15—C14	1.406 (3)
Cd1—N2	2.4153 (17)	C13—H13	0.9300
Cd1—N2^i^	2.4153 (17)	C13—C14	1.366 (2)
S1—O3	1.4335 (15)	C14—H14	0.9300
S1—O2	1.4601 (15)	C12—C11	1.398 (3)
S1—O1	1.4556 (15)	C6—C1	1.390 (3)
S1—C16	1.7765 (16)	C6—C5	1.388 (3)
N3—C7	1.446 (2)	C1—C2	1.381 (3)
N3—H3A	0.86 (3)	C2—H2	0.9300
N3—H3B	0.81 (3)	C2—C3	1.386 (3)
N1—C1	1.437 (2)	C8—H8	0.9300
N1—H1A	0.88 (3)	C8—C9	1.392 (3)
N1—H1B	0.83 (3)	C5—H5	0.9300
N2—C6	1.434 (2)	C5—C4	1.380 (4)
N2—H2A	0.81 (3)	C11—H11	0.9300
N2—H2B	0.87 (3)	C11—C10	1.370 (4)
C17—C17^ii^	1.423 (3)	C3—H3	0.9300
C17—C16	1.440 (2)	C3—C4	1.380 (4)
C17—C13^ii^	1.415 (2)	C4—H4	0.9300
C7—C12	1.393 (3)	C9—H9	0.9300
C7—C8	1.379 (3)	C9—C10	1.376 (4)
N4—C12	1.403 (3)	C10—H10	0.9300
N4—H4A	0.91 (3)		
			
N3^i^—Cd1—N3	80.58 (7)	H4A—N4—H4B	106 (3)
N1^i^—Cd1—N3	101.57 (6)	C16—C15—H15	119.8
N1^i^—Cd1—N3^i^	92.10 (6)	C16—C15—C14	120.34 (15)
N1—Cd1—N3^i^	101.57 (6)	C14—C15—H15	119.8
N1—Cd1—N3	92.10 (6)	C17—C16—S1	121.00 (12)
N1^i^—Cd1—N1	162.11 (10)	C15—C16—S1	117.74 (12)
N1—Cd1—N2	70.41 (6)	C15—C16—C17	121.26 (15)
N1—Cd1—N2^i^	96.90 (6)	C17^ii^—C13—H13	119.3
N1^i^—Cd1—N2	96.89 (6)	C14—C13—C17^ii^	121.44 (16)
N1^i^—Cd1—N2^i^	70.41 (6)	C14—C13—H13	119.3
N2—Cd1—N3	94.02 (6)	C15—C14—H14	119.9
N2—Cd1—N3^i^	170.30 (6)	C13—C14—C15	120.15 (16)
N2^i^—Cd1—N3^i^	94.02 (6)	C13—C14—H14	119.9
N2^i^—Cd1—N3	170.30 (6)	C7—C12—N4	120.60 (17)
N2—Cd1—N2^i^	92.41 (9)	C7—C12—C11	118.13 (19)
O3—S1—O2	113.53 (10)	C11—C12—N4	121.22 (19)
O3—S1—O1	113.92 (10)	C1—C6—N2	118.20 (16)
O3—S1—C16	107.01 (9)	C5—C6—N2	122.42 (19)
O2—S1—C16	105.95 (8)	C5—C6—C1	119.26 (18)
O1—S1—O2	110.66 (9)	C6—C1—N1	117.96 (17)
O1—S1—C16	105.00 (8)	C2—C1—N1	121.76 (19)
Cd1—N3—H3A	105.4 (16)	C2—C1—C6	120.12 (19)
Cd1—N3—H3B	99.6 (19)	C1—C2—H2	119.8
C7—N3—Cd1	126.76 (11)	C1—C2—C3	120.4 (2)
C7—N3—H3A	110.4 (16)	C3—C2—H2	119.8
C7—N3—H3B	111.3 (18)	C7—C8—H8	119.9
H3A—N3—H3B	100 (2)	C7—C8—C9	120.1 (2)
Cd1—N1—H1A	102.8 (16)	C9—C8—H8	119.9
Cd1—N1—H1B	114 (2)	C6—C5—H5	119.8
C1—N1—Cd1	107.87 (12)	C4—C5—C6	120.3 (2)
C1—N1—H1A	110.1 (17)	C4—C5—H5	119.8
C1—N1—H1B	111 (2)	C12—C11—H11	119.6
H1A—N1—H1B	111 (3)	C10—C11—C12	120.8 (2)
Cd1—N2—H2A	100 (2)	C10—C11—H11	119.6
Cd1—N2—H2B	116.2 (19)	C2—C3—H3	120.2
C6—N2—Cd1	105.88 (12)	C4—C3—C2	119.5 (2)
C6—N2—H2A	113 (2)	C4—C3—H3	120.2
C6—N2—H2B	110.7 (19)	C5—C4—H4	119.8
H2A—N2—H2B	111 (3)	C3—C4—C5	120.4 (2)
C17^ii^—C17—C16	117.68 (18)	C3—C4—H4	119.8
C13^ii^—C17—C17^ii^	119.12 (18)	C8—C9—H9	120.3
C13^ii^—C17—C16	123.20 (15)	C10—C9—C8	119.3 (2)
C12—C7—N3	119.18 (16)	C10—C9—H9	120.3
C8—C7—N3	119.98 (17)	C11—C10—C9	120.8 (2)
C8—C7—C12	120.84 (17)	C11—C10—H10	119.6
C12—N4—H4A	113.3 (18)	C9—C10—H10	119.6
C12—N4—H4B	110 (3)		
			
Cd1—N3—C7—C12	−62.9 (2)	C17^ii^—C13—C14—C15	0.8 (3)
Cd1—N3—C7—C8	116.43 (18)	C7—C12—C11—C10	0.0 (4)
Cd1—N1—C1—C6	33.6 (2)	C7—C8—C9—C10	0.2 (4)
Cd1—N1—C1—C2	−141.87 (17)	N4—C12—C11—C10	177.5 (3)
Cd1—N2—C6—C1	−31.2 (2)	C16—C15—C14—C13	−0.6 (3)
Cd1—N2—C6—C5	144.82 (18)	C13^ii^—C17—C16—S1	1.4 (2)
O3—S1—C16—C17	−179.81 (14)	C13^ii^—C17—C16—C15	−179.75 (17)
O3—S1—C16—C15	1.27 (17)	C14—C15—C16—S1	178.96 (14)
O2—S1—C16—C17	−58.37 (15)	C14—C15—C16—C17	0.0 (3)
O2—S1—C16—C15	122.71 (15)	C12—C7—C8—C9	0.6 (3)
O1—S1—C16—C17	58.79 (15)	C12—C11—C10—C9	0.7 (5)
O1—S1—C16—C15	−120.12 (15)	C6—C1—C2—C3	0.7 (3)
N3—C7—C12—N4	1.2 (3)	C6—C5—C4—C3	0.0 (4)
N3—C7—C12—C11	178.65 (19)	C1—C6—C5—C4	0.9 (3)
N3—C7—C8—C9	−178.7 (2)	C1—C2—C3—C4	0.1 (4)
N1—C1—C2—C3	176.0 (2)	C2—C3—C4—C5	−0.5 (4)
N2—C6—C1—N1	−0.6 (3)	C8—C7—C12—N4	−178.1 (2)
N2—C6—C1—C2	174.92 (19)	C8—C7—C12—C11	−0.7 (3)
N2—C6—C5—C4	−175.1 (2)	C8—C9—C10—C11	−0.8 (5)
C17^ii^—C17—C16—S1	−178.68 (15)	C5—C6—C1—N1	−176.70 (19)
C17^ii^—C17—C16—C15	0.2 (3)	C5—C6—C1—C2	−1.2 (3)

Symmetry codes: (i) −*x*+1, *y*, −*z*+3/2; (ii) −*x*+1, −*y*+2, −*z*+1.

### Hydrogen-bond geometry (Å, º)

**Table:**

*D*—H···*A*	*D*—H	H···*A*	*D*···*A*	*D*—H···*A*
N3—H3*A*···O2	0.86 (3)	2.36 (3)	3.185 (2)	160 (2)
N2—H2*A*···O2^iii^	0.81 (3)	2.30 (3)	3.046 (3)	154 (3)
N1—H1*B*···O1	0.83 (3)	2.18 (3)	2.955 (2)	156 (3)
N3—H3*B*···O2^i^	0.81 (3)	2.37 (3)	3.097 (2)	150 (2)
N4—H4*A*···O1^i^	0.91 (3)	2.11 (3)	3.021 (3)	176 (3)

Symmetry codes: (i) −*x*+1, *y*, −*z*+3/2; (iii) *x*, *y*−1, *z*.
